# Predictors of Depression, Anxiety, and Stress Symptoms Among Older Adults in Rural North India

**DOI:** 10.7759/cureus.115559

**Published:** 2026-08-31

**Authors:** Akshay Kumar, Prem Prakash Bharati, Divyansh Singh, Kajal Kureel, Shivali Singh, Pankaj Jain, Sandip Kumar

**Affiliations:** 1 Department of Community Medicine, Uttar Pradesh University of Medical Sciences, Etawah, IND; 2 Department of Community Medicine, King George's Medical University, Lucknow, IND; 3 Department of Anaesthesiology, Dr. Bhimrao Ramji Ambedkar, Government Medical College, Kannauj, IND

**Keywords:** anxiety, community-based study, dass-21, depression, older adults, predictors, rural india, stress

## Abstract

Background: Mental health problems among older adults are an important and underrecognized public health concern in India. Depression, anxiety, and stress in later life are influenced by demographic, social, behavioural, and clinical factors, and identifying their independent predictors is essential for designing targeted preventive strategies.

Objectives: This study aims to estimate the burden of depression, anxiety, and stress among older adults in rural North India and to identify independent predictors of depression, anxiety, and stress symptoms.

Methods: A community-based cross-sectional study was conducted among 384 older adults aged 60 years and above residing in rural districts of North India. Participants were selected through multistage random sampling from three rural blocks, and door-to-door household interviews were carried out. Data were collected using a pretested semi-structured questionnaire. Depression, anxiety, and stress were assessed using the Depression, Anxiety, and Stress Scale (DASS-21). Bivariate analysis was performed using the chi-square test, followed by multivariable binary logistic regression to identify factors independently associated with depression, anxiety, and stress.

Results: The sample included 53.4% males and 46.6% females. Most participants were aged 60-65 years (64.3%), 64.1% were illiterate, 65.9% were married, and 60.9% belonged to lower socioeconomic strata. In this study, depression was present in 98 participants (25.5%), anxiety in 134 participants (34.9%), and stress in 100 participants (26.0%). This study also demonstrated strong positive correlations between depression, anxiety, and stress scores. Depression showed a strong positive correlation with anxiety (ρ = 0.865, p < 0.001) and stress (ρ = 0.852, p < 0.001). The strongest correlation was observed between anxiety and stress scores (ρ = 0.835, p < 0.001). In the final multivariable models, sleep difficulty, social isolation, chronic morbidity, and physical inactivity emerged as factors independently associated with psychological distress.

Conclusion: Psychological distress is common among older adults in rural districts of North India, and sleep difficulty, chronic morbidity, social isolation, and physical inactivity are key predictors. Community-based geriatric mental health programs should incorporate routine screening for these factors and link high-risk older adults to primary care and social support services.

## Introduction

Population ageing is emerging as one of the most significant public health challenges globally and in India [1]. Improvements in healthcare, nutrition, and living conditions have led to increased life expectancy, resulting in a rapidly growing population of older adults requiring long-term health and social support [2]. Ageing is often accompanied by biological decline, multimorbidity, reduced functional capacity, bereavement, economic dependency, and changing social roles, all of which may adversely affect psychological well-being. Consequently, depression, anxiety, and stress have become increasingly important mental health concerns among older adults [3]. Despite their high prevalence, these conditions frequently remain underrecognized and undertreated because their manifestations in older adults may be atypical and masked by physical symptoms. Mental health in later life is influenced not only by physical health but also by social and environmental factors [4,5]. Social isolation, widowhood, low socioeconomic status, physical inactivity, sleep disturbances, and chronic medical conditions have been consistently identified as important determinants of psychological distress among older adults [4-7]. In rural settings, these vulnerabilities may be further compounded by lower educational attainment, limited health literacy, inadequate social support systems, and restricted access to mental health services. Since many of these factors are potentially modifiable, identifying their contribution to mental health outcomes is essential for planning effective public health interventions and primary healthcare strategies.

The global demographic transition towards an ageing population has been unprecedented. In 2020, approximately one billion people worldwide were aged 60 years or above, and this number is projected to increase to 1.4 billion by 2030 and 2.1 billion by 2050 [8]. During the same period, the proportion of older adults in the global population is expected to increase substantially, reflecting the rapid pace of population ageing [8]. Furthermore, the number of older adults aged 80 years and above is expected to triple by 2050, reaching nearly 426 million worldwide [8]. According to the World Health Organization, one in every six individuals globally will be aged 60 years or older by 2030 [1,9]. This demographic shift has important implications for health systems, particularly with regard to the growing burden of chronic diseases and mental health disorders among older adults. Mental disorders represent a substantial proportion of disability among older adults. According to the Global Burden of Disease Study, depression is among the leading contributors to disability worldwide and is expected to remain a major public health concern in developing countries [10]. Approximately 14% of individuals aged 60 years and above experience a mental health disorder, and mental illnesses account for a significant proportion of years lived with disability in this age group. Among these conditions, depression remains the most common mental disorder, affecting millions of older adults worldwide [3,11].

Indian scenario

India is currently undergoing a rapid demographic transition characterized by a steadily increasing older adults population [8,9]. Although the proportion of older adults is rising, comprehensive evidence regarding the burden and determinants of mental health disorders among older adults remains limited [3,10]. Depression has emerged as a major public health concern due to its association with impaired quality of life, functional decline, increased healthcare utilization, and elevated risk of suicidal behaviour [3,10,11]. Projections suggest that the burden of depression among older adults in India is likely to increase substantially in the coming decades, posing significant challenges to healthcare systems and social support structures [8,9].

Depression among older adults is often underdiagnosed because of its heterogeneous clinical presentation and frequent coexistence with chronic physical illnesses [6,7]. Older adults experiencing depression commonly exhibit reduced physical functioning, diminished independence, and poorer overall health outcomes compared with their non-depressed counterparts [6,7]. Several Indian and international studies have documented the prevalence of depression, anxiety, and stress among older adults [3,12-14]. However, relatively few community-based studies have simultaneously assessed depression, anxiety, and stress using a standardized assessment instrument and examined the independent factors associated with each outcome among older adults in rural Indian settings [3,7,14]. This represents an important evidence gap, particularly in rural North Indian populations, where social, socioeconomic, behavioural, and health-related factors may differ from those observed in other settings. Previous epidemiological studies conducted in rural India have reported a considerable burden of depression among older adults; for example, a community-based study from rural South India reported a prevalence of 12.7% [8]. Therefore, this study was undertaken to assess the burden of depression, anxiety, and stress among older adults residing in a rural study area and to identify the factors independently associated with these psychological conditions.

## Materials and methods

Study design and setting

This community-based cross-sectional study was conducted in the rural field practice area of a tertiary care teaching institution. A multistage random sampling strategy was adopted for participant selection. Initially, three administrative blocks were randomly selected from a total of eight administrative blocks in the study area. Subsequently, eight villages were randomly selected from each selected block, resulting in a total of 24 selected villages. From each selected village, 16 households were randomly selected, resulting in a total of 384 households. Data were collected through face-to-face interviews with eligible older adults aged 60 years and above using a pretested semi-structured questionnaire. One eligible older adult was included from each selected household. In households with more than one eligible older adult, one participant was selected randomly. If a selected household was locked, unoccupied, or had no eligible participant available at the time of the visit, an adjacent household was approached as a replacement. Depression, anxiety, and stress symptoms were assessed using the Depression Anxiety Stress Scale-21 (DASS-21) [15]. Descriptive statistics were computed for all study variables, and associations were evaluated using the chi-square test. Multivariable binary logistic regression was used to identify independent predictors of depression, anxiety, and stress. Statistical significance was determined at a p-value of <0.05.

Inclusion and exclusion criteria

Participants eligible for inclusion in the study were older adults aged 60 years and above who were permanent residents of the study area and had been residing there for at least one year preceding the survey. Older adults who were seriously ill and unable to participate in the interview at the time of data collection, those with severe cognitive impairment or psychiatric illness affecting reliable communication, and those with severe hearing or speech impairment that prevented administration of the questionnaire were excluded from the study. Written informed consent was obtained from all participants before enrolment in the study.

Data collection tools

Data were collected using a pretested semi-structured questionnaire, which comprised two components: sociodemographic details of older adults and assessment of depression, anxiety, and stress using the DASS-21 scale [15]. The questionnaire was administered through face-to-face interviews by trained researchers to ensure uniformity and accuracy of data collection.

Study population

The study population consisted of older adults aged 60 years and above residing in the rural areas of the North Indian district.

Sample size

The sample size was calculated using the standard formula for estimating prevalence in cross-sectional studies: n = (Z² × p × q) / d² [16], where n = required sample size, Z = standard normal variate at 95% confidence level (1.96), p = estimated prevalence of depression among older adults, q = 1 − p, and d = allowable error (absolute precision). The prevalence (p) of depression among the older adults was assumed to be 50% to obtain the maximum sample size. Thus, n = (1.96)² × (0.5 × 0.5) / (0.05)², n = 384.16. Thus, the minimum required sample size was 384 older adults.

Data collection

Data collection was carried out through a multistage random sampling approach. Stage 1 - Selection of administrative blocks: Three administrative blocks were randomly selected from a total of eight administrative blocks in the study area. Stage 2 - Selection of villages: The three selected administrative blocks comprised a total of 257 villages. Eight villages were randomly selected from each selected block, resulting in a total of 24 selected villages. Stage 3 - Selection of households: Sixteen households were randomly selected from each selected village, resulting in a total of 384 selected households. Stage 4 - Selection of study participants: Trained field investigators visited the selected households and conducted face-to-face interviews with eligible older adults aged ≥60 years using the pretested semi-structured questionnaire. One eligible older adult was selected from each household; where more than one eligible older adult was present, one participant was selected randomly. If a selected household was found to be locked, unoccupied, or had no eligible participant available at the time of the visit, an adjacent household was approached as a replacement. A total of 384 older adults were successfully enrolled in the study, resulting in a 100% response rate. Complete data were available for all enrolled participants, and no missing values were present in the final dataset; therefore, no imputation or other missing-data handling procedures were required. Data were analyzed using IBM SPSS Statistics for Windows, Version 25 (Released 2017; IBM Corp., Armonk, New York) [17].

Operational definitions

Depression, Anxiety, and Stress

Depression, anxiety, and stress symptoms were assessed using the DASS-21 [15]. The original English version of the DASS-21 was translated into Hindi by the investigators. The Hindi version was independently back-translated into English by a bilingual individual to assess conceptual and linguistic equivalence. The translated version was pilot-tested among 50 older adults to assess clarity, comprehensibility, and acceptability. These participants were not included in the final study sample. Formal psychometric validation and internal consistency assessment (Cronbach's alpha) of the study-specific Hindi translation were not performed. The scale consists of 21 items, with seven items each assessing depression, anxiety, and stress. Scores for each domain were calculated according to standard DASS-21 scoring guidelines by summing the item scores and multiplying the total by two. Participants with mild, moderate, severe, or extremely severe scores were categorized as having depression, anxiety, or stress symptoms, respectively, whereas those with normal scores were categorized as not having the respective symptoms.

Social Isolation

Social isolation was assessed by asking participants whether they experienced feelings of loneliness or social isolation. Participants reporting occasional, sometimes frequent, or everyday feelings of loneliness/social isolation were categorized as socially isolated, whereas those reporting no such feelings were categorized as not socially isolated.

Sleep Difficulty

Sleep difficulty was assessed using a self-reported question: “Did you experience any sleep difficulties?” Participants answering “Yes” were classified as having sleep difficulty, whereas those answering “No” were classified as not having sleep difficulty. Sleep duration during the previous two weeks was additionally recorded as less than 4 hours, 4-5 hours, 6 hours, 7-8 hours, and 9 or more hours in a 24-hour period.

Physical Activity

Participants were classified as physically active if they reported engagement in household work, labour work, farm work, walking, cycling, or any combination of these activities. Participants reporting no such activity were classified as physically inactive.

Chronic Morbidity

Chronic morbidity was defined as the presence of one or more self-reported physician-diagnosed chronic medical conditions, including hypertension, diabetes mellitus, chronic obstructive pulmonary disease (COPD), arthritis, cardiovascular disease, or other long-term illnesses requiring ongoing treatment. Participants reporting at least one chronic medical condition were categorized as having chronic morbidity.

Socioeconomic Status

Socioeconomic status was assessed using the Modified B.G. Prasad Socioeconomic Classification [18]. Participants were categorized into socioeconomic classes according to the prevailing income-based classification. For regression analysis, categories were grouped as lower socioeconomic status and upper/middle socioeconomic status.

Data Management and Statistical Analysis

Data were entered into Microsoft Excel version 2019 (Microsoft Corporation, Redmond, Washington) and analyzed using IBM SPSS Statistics for Windows, Version 25 (Released 2017; IBM Corp., Armonk, New York) [17]. Descriptive statistics were used to summarize participant characteristics and estimate the prevalence of depression, anxiety, and stress. Associations between categorical variables were assessed using the chi-square test. Spearman's rank correlation coefficient was used to assess the correlations between depression, anxiety, and stress scores. Variables demonstrating statistical significance in bivariate analysis (p<0.05), together with epidemiologically relevant covariates identified from previous literature, were entered into multivariable binary logistic regression models using the enter method to identify independent predictors of depression, anxiety, and stress. Adjusted odds ratios (AORs) with 95% confidence intervals (CIs) were reported. Statistical significance was considered at p<0.05.

Ethical consideration

The study was conducted after obtaining written ethical approval from the Institutional Ethics Committee (IEC) of King George's Medical University, Lucknow, Uttar Pradesh (Letter No. 2796/Ethics/2023, Ref. Code XVI-PGTSC-IIA/P56). Written informed consent was obtained from all participants prior to enrolment. The confidentiality and anonymity of the participants were strictly maintained throughout the study.

Outcome variables

The primary outcome variables were depression, anxiety, and stress symptoms. Depression, anxiety, and stress were dichotomized into “present” (mild, moderate, severe, and extremely severe) and “absent” (normal) categories for logistic regression analysis.

Predictor variables

Candidate predictor variables included age, sex, marital status, education, socioeconomic status, family type, social isolation, physical activity, sleep difficulty, substance use, and chronic morbidity. For analysis, marital status was dichotomized as widow/widower versus non-widow/widower.

## Results

Table 1 shows that a total of 384 older adults participated in the study, including 205 (53.4%) males and 179 (46.6%) females. Most participants belonged to the 60-65-year-old age group and were married. The majority were living in joint or nuclear families. Educational attainment was generally low, with a large proportion of participants being illiterate. Most participants belonged to the lower and lower-middle socioeconomic classes according to the Modified B.G. Prasad classification, indicating that the study population predominantly consisted of socioeconomically disadvantaged rural older adults.

**Table 1:** Sociodemographic characteristics of the study participants (N=384) *According to the Modified B.G. Prasad Socioeconomic Classification updated in October 2023 [18].

Variable	Female		Male	
	N	(%)	N	(%)
Gender	179	46.6	205	53.4
Age group (in years)				
60–65	122	49.4	125	50.6
66–70	29	38.7	46	61.3
71–75	17	45.9	20	54.1
>75	11	44.0	14	56.0
Marital status				
Unmarried	0	0	4	100
Married	96	37.9	157	62.1
Divorced	4	80	1	20
Widow/widower	80	65	43	35
Type of Family				
Joint family	82	44.6	102	55.4
Nuclear family	86	48.3	92	51.7
Only with wife/husband	8	50	8	50
Living alone	3	50	3	50
Educational Qualification				
Illiterate	153	62.2	93	37.8
Less than primary school	9	33.3	18	66.7
Primary school	8	25.8	23	74.2
Middle school	4	18.2	18	81.8
High school	3	20	12	80
Intermediate	2	7.7	24	92.3
Graduate degree and above	0	0.0	17	100
Socioeconomic status*				
Upper class	4	23.5	13	76.5
Upper middle class	4	36.4	7	63.6
Middle class	12	37.5	20	62.5
Lower middle class	44	48.9	46	51.1
Lower class	115	49.1	119	50.9

Table 2 suggests that among the 384 participants, depressive symptoms were present in 98 (25.5%; 95% CI: 21.2-30.2%), anxiety symptoms in 134 (34.9%; 95% CI: 30.1-39.9%), and stress symptoms in 100 (26.0%; 95% CI: 21.7-30.7%). Anxiety symptoms were the most frequently observed, followed by stress and depressive symptoms. Overall, these findings indicate a substantial burden of psychological symptoms among older adults in the study population.

**Table 2:** Prevalence of depressive, anxiety, and stress symptoms among older adults (N = 384)

Outcome	n	%	95% CI
Depressive symptoms	98	25.5	21.2–30.2
Anxiety symptoms	134	34.9	30.1–39.9
Stress symptoms	100	26.0	21.7–30.7

In Table 3, Spearman's rank correlation analysis demonstrated significant positive correlations among the three DASS-21 domains. Depression showed a strong positive correlation with anxiety (ρ = 0.865, p < 0.001) and stress (ρ = 0.852, p < 0.001), while anxiety showed a strong positive correlation with stress (ρ = 0.835, p < 0.001). These findings indicate substantial shared variance among the three psychological symptom domains and should be considered when interpreting the findings from the separate outcome-specific regression models.

**Table 3:** Spearman's rank correlation between depression, anxiety, and stress scores among the older adult population (N = 384) *Statistical significance was considered at p < 0.05.

Variables	Spearman's Rank Correlation Coefficient (ρ)	p-value
Depression vs. Anxiety	0.865	<0.001*
Depression vs. Stress	0.852	<0.001*
Anxiety vs. Stress	0.835	<0.001*

In Table 4, bivariate analysis showed that female sex, widowhood, illiteracy, social isolation, sleep difficulty, and chronic morbidity were significantly associated with depression. Anxiety was significantly associated with female sex, widowhood, illiteracy, social isolation, sleep difficulty, and chronic morbidity. Stress was significantly associated with female sex, widowhood, social isolation, sleep difficulty, physical inactivity, and chronic morbidity. Age ≥70 years was not significantly associated with depression, anxiety, or stress (p=0.184, p=0.119, and p=0.787, respectively). Social isolation and sleep difficulty demonstrated the strongest associations across all three psychological outcomes.

**Table 4:** Bivariate analysis of factors associated with depression, anxiety, and stress in older adult population (N = 384) *Statistical significance was considered at p < 0.05. **Widow/widower represents a dichotomous marital-status variable (widow/widower vs. non-widow/widower). Abbreviations: χ² = Pearson chi-square statistic; df = degrees of freedom; Phi = Phi coefficient (effect size for 2×2 contingency tables).

Variable	Depression χ²	df	Phi	p-value	Anxiety χ²	df	Phi	p-value	Stress χ²	df	Phi	p-value
Female sex	11.29	1	0.171	<0.001*	8.44	1	0.148	0.004	7.04	1	0.135	0.008
Age ≥70 years	1.77	1	0.068	0.184	2.44	1	0.080	0.119	0.07	1	0.014	0.787
Widow/widower**	20.17	1	0.229	<0.001*	16.06	1	0.205	<0.001*	7.87	1	0.143	0.005*
Illiteracy	8.88	1	0.152	0.003*	8.62	1	0.150	0.003*	2.07	1	0.073	0.150
Lower socioeconomic status	0.01	1	0.005	0.920	0.37	1	0.031	0.543	0.19	1	0.022	0.660
Social isolation	57.51	1	0.387	<0.001*	48.60	1	0.356	<0.001*	69.41	1	0.425	<0.001*
Sleep difficulty	71.03	1	0.430	<0.001*	65.34	1	0.413	<0.001*	48.86	1	0.357	<0.001*
Physical inactivity	1.87	1	0.070	0.171	3.75	1	0.099	0.053	8.56	1	0.149	0.003*
Chronic morbidity	11.43	1	0.173	<0.001*	14.66	1	0.195	<0.001*	11.58	1	0.174	<0.001*

In Table 5, multivariable logistic regression identified sleep difficulty, social isolation, widowhood, and chronic morbidity as significant independent predictors of depression. Participants with sleep difficulty had more than five-fold higher odds of depression (AOR = 5.21, 95% CI: 2.66-10.24, p < 0.001). Social isolation (AOR = 2.11, 95% CI: 1.09-4.06, p = 0.026), widowhood (AOR = 2.02, 95% CI: 1.09-3.74, p = 0.026), and chronic morbidity (AOR = 2.30, 95% CI: 1.30-4.08, p = 0.004) were also independently associated with depression.

**Table 5:** Multivariable logistic regression for predictors of depression in older adult population (N = 384) *Statistical significance was considered at p < 0.05. Contingency-table counts are presented as exposed + outcome present / exposed + outcome absent; reference + outcome present / reference + outcome absent. Outcome present includes mild, moderate, severe, and extremely severe categories; outcome absent indicates normal. Abbreviations: OR = odds ratio; CI = confidence interval; Ref = reference category.

Variable (reference category)	Contingency table counts	Unadjusted OR	95% CI (unadjusted)	p-value (unadjusted)	Adjusted OR	95% CI (adjusted)	p-value (adjusted)
Female sex (Ref: Male)	60/119; 38/167	2.22	1.39–3.54	<0.001	0.88	0.46–1.66	0.684
Age ≥70 years (Ref: Age <70 years)	38/74; 60/212	1.81	1.12–2.95	0.016	0.82	0.44–1.52	0.521
Widow/Widower (Ref: Non-widow/widower)	49/73; 49/213	2.92	1.81–4.70	<0.001	2.02	1.09–3.74	0.026*
Illiteracy (Ref: Literate)	75/171; 23/115	2.19	1.30–3.70	0.003	0.96	0.48–1.93	0.906
Lower socioeconomic status (Ref: Upper/middle socioeconomic status)	59/175; 39/111	0.96	0.60–1.53	0.863	0.71	0.40–1.26	0.240
Social isolation (Ref: No social isolation)	66/71; 32/215	6.25	3.79–10.30	<0.001	2.11	1.09–4.06	0.026*
Sleep difficulty (Ref: No sleep difficulty)	71/71; 27/215	7.96	4.74–13.37	<0.001	5.21	2.66–10.24	<0.001*
Physical inactivity (Ref: Physically active)	12/22; 86/264	1.67	0.80–3.52	0.175	1.72	0.73–4.04	0.213
Chronic morbidity (Ref: No chronic morbidity)	52/100; 46/186	2.10	1.32–3.35	0.002	2.30	1.30–4.08	0.004*

In Table 6, multivariable logistic regression demonstrated that sleep difficulty, social isolation, chronic morbidity, and lower socioeconomic status were significant independent predictors of anxiety. Participants reporting sleep difficulty had 4.65 times higher odds of anxiety compared with those without sleep difficulty (AOR = 4.65, 95% CI: 2.52-8.60, p < 0.001). Social isolation was associated with increased odds of anxiety (AOR = 1.89, 95% CI: 1.03-3.47, p = 0.039), while chronic morbidity was associated with higher odds of anxiety (AOR = 2.38, 95% CI: 1.43-3.97, p = 0.001). Lower socioeconomic status was associated with significantly lower odds of anxiety compared with upper/middle socioeconomic status (AOR = 0.42, 95% CI: 0.21-0.84, p = 0.015), despite the absence of a significant association in the bivariate analysis. Female sex, age ≥70 years, widowhood, illiteracy, and physical inactivity were not independently associated with anxiety after adjustment for other covariates.

**Table 6:** Multivariable logistic regression for predictors of anxiety in older adult population (N = 384) *Statistical significance was considered at p < 0.05. Contingency-table counts are presented as exposed + outcome present / exposed + outcome absent; reference + outcome present / reference + outcome absent. Outcome present includes mild, moderate, severe, and extremely severe categories; outcome absent indicates normal. Abbreviations: OR = odds ratio; CI = confidence interval; Ref = reference category.

Variable (reference category)	Contingency table counts	Unadjusted OR	95% CI (unadjusted)	p-value (unadjusted)	Adjusted OR	95% CI (adjusted)	p-value (adjusted)
Female sex (Ref: Male)	76/103; 58/147	1.87	1.22–2.86	0.004	0.84	0.47–1.48	0.536
Age ≥70 years (Ref: Age <70 years)	51/61; 83/189	1.90	1.21–2.99	0.005	0.92	0.52–1.63; p=0.769	0.769
Widow/Widower (Ref: Non-widow/widower)	60/62; 74/188	2.46	1.57–3.84	<0.001	1.72	0.97–3.05	0.062
Illiteracy (Ref: Literate)	99/147; 35/103	1.98	1.25–3.14	0.004	1.09	0.59–2.02	0.772
Lower socioeconomic status (Ref: Upper/middle socioeconomic status)	79/155; 55/95	0.88	0.57–1.35	0.560	0.42	0.21–0.84	0.015*
Social isolation (Ref: No social isolation)	79/58; 55/192	4.75	3.02–7.48	<0.001	1.89	1.03–3.47	0.039*
Sleep difficulty (Ref: No sleep difficulty)	86/56; 48/194	6.21	3.91–9.85	<0.001	4.65	2.52–8.60	<0.001*
Physical inactivity (Ref: Physically active)	17/17; 117/233	1.99	0.98–4.04	0.057	2.18	0.98–4.84	0.056
Chronic morbidity (Ref: No chronic morbidity)	70/82; 64/168	2.24	1.46–3.44	<0.001	2.38	1.43–3.97	0.001*

In Table 7, multivariable logistic regression identified social isolation, sleep difficulty, physical inactivity, and chronic morbidity as significant independent predictors of stress. Social isolation was the strongest predictor, with socially isolated participants having more than five-fold higher odds of stress (AOR = 5.47, 95% CI: 2.77-10.79, p < 0.001). Physical inactivity was associated with higher odds of stress (AOR = 4.25, 95% CI: 1.87-9.70, p = 0.001), as was sleep difficulty (AOR = 2.73, 95% CI: 1.39-5.38, p = 0.004). Chronic morbidity was also independently associated with stress (AOR = 2.67, 95% CI: 1.50-4.74, p = 0.001).

**Table 7:** Multivariable logistic regression for predictors of stress in older adult population (N = 384) *Statistical significance was considered at p < 0.05. Contingency-table counts are presented as exposed + outcome present / exposed + outcome absent; reference + outcome present / reference + outcome absent. Outcome present includes mild, moderate, severe, and extremely severe categories; outcome absent indicates normal. Abbreviations: OR = odds ratio; CI = confidence interval; Ref = reference category.

Variable (reference category)	Contingency table counts	Unadjusted OR	95% CI (unadjusted)	p-value (unadjusted)	Adjusted OR	95% CI (adjusted)	p-value (adjusted)
Female sex (Ref: Male)	58/121; 42/163	1.86	1.17–2.95	0.008	0.98	0.52–1.85	0.946
Age ≥70 years (Ref: Age <70 years)	35/77; 65/207	1.45	0.89–2.36	0.137	0.78	0.41–1.45	0.429
Widow/Widower (Ref: Non-widow/widower)	43/79; 57/205	1.96	1.22–3.14	0.005	1.18	0.63–2.22	0.605
Illiteracy (Ref: Literate)	70/176; 30/108	1.43	0.88–2.34	0.151	0.56	0.28–1.12	0.099
Lower socioeconomic status (Ref: Upper/middle socioeconomic status)	59/175; 41/109	0.90	0.56–1.43	0.644	0.47	0.22–1.01	0.052
Social isolation (Ref: No social isolation)	70/67; 30/217	7.56	4.55–12.56	<0.001	5.47	2.77–10.79	<0.001*
Sleep difficulty (Ref: No sleep difficulty)	66/76; 34/208	5.31	3.25–8.67	<0.001	2.73	1.39–5.38	0.004*
Physical inactivity (Ref: Physically active)	16/18; 84/266	2.81	1.37–5.76	0.005	4.25	1.87–9.70	0.001*
Chronic morbidity (Ref: No chronic morbidity)	53/99; 47/185	2.11	1.33–3.35	0.002	2.67	1.50–4.74; p=0.001	0.001*

## Discussion

This community-based study assessed the burden and predictors of depression, anxiety, and stress among older adults residing in rural areas. The findings revealed that depression, anxiety, and stress symptoms affected 25.5%, 34.9%, and 26.0% of the study participants, respectively. Furthermore, strong positive correlations were observed between depression, anxiety, and stress scores, indicating that these psychological conditions frequently coexist in older adults. These findings underscore the substantial burden of mental health problems among older adults and highlight the need for integrated screening and intervention strategies. As this was a cross-sectional study, the observed associations should be interpreted as statistical associations rather than causal relationships, and temporal directionality between the predictors and psychological outcomes cannot be established.

The prevalence of depressive symptoms observed in this study (25.5%) was comparable to the pooled prevalence of 19.2% reported in a recent meta-analysis by Jalali et al. and is also close to the 24.2% prevalence reported by Lugova et al. among older adults [3,12]. However, the prevalence observed in the current study was lower than that reported by Zenebe et al. (31.7%), Raeisvandi et al. (45.5%), Mathew et al. (49%), and Sharma et al. (60.8%) [14,19-21]. These differences may reflect variations in study settings, sociocultural characteristics, screening instruments, and the presence of chronic illnesses among study populations. Nevertheless, the prevalence observed in this study clearly demonstrates that depression remains an important public health problem among older adults living in rural communities.

The prevalence of anxiety in this study was 34.9%, which is substantially higher than the pooled prevalence of 16.5% reported by Jalali et al. and the 21.1% prevalence observed by Xie et al. among older adults [3,13]. However, it is comparable to findings reported by Lugova et al. (36.3%) and Raeisvandi et al. (35.5%) [12,19]. The relatively high prevalence of anxiety symptoms in this study may reflect increasing health-related concerns, financial dependency, social isolation, fear of disability, and uncertainty regarding future health among older adults. Rural populations often face additional barriers to healthcare access and mental health services, which may be associated with a greater burden of anxiety symptoms.

Similarly, the prevalence of stress symptoms in this study (26.0%) was higher than the pooled prevalence reported by Jalali et al. (13.9%) and Lugova et al. (20.6%) but lower than that reported by Raeisvandi et al. (40.2%) [3,12,19]. These findings suggest that stress represents a significant yet often overlooked mental health concern among older adults. The presence of chronic illnesses, social disconnection, reduced physical functioning, and increasing dependence on family members may be associated with greater levels of stress in later life.

An important finding of this study was the strong positive correlation between depression, anxiety, and stress scores. Depression showed significant positive correlations with anxiety (ρ = 0.865) and stress (ρ = 0.852), while anxiety and stress demonstrated the strongest correlation (ρ = 0.835), all p < 0.001. These findings indicate considerable overlap between emotional disorders in old age and support the concept that depression, anxiety, and stress often coexist rather than occur independently. Although separate multivariable logistic regression models were used for each outcome, the observed intercorrelations indicate substantial shared variance among the DASS-21 subscales, which should be considered when interpreting the independent associations identified in the regression models. Similar observations have been reported by Dow et al., who reported significant associations between depression and anxiety symptoms among older adults [22]. Lin et al. also highlighted the importance of social relationships for psychological well-being among older adults [23]. Such findings emphasize the importance of comprehensive mental health screening and consideration of the broader social context rather than focusing on a single psychological condition.

In the multivariable logistic regression model for depression, sleep difficulty showed the strongest independent association. Older adults reporting sleep difficulty had more than five times higher odds of depression than those without sleep problems. This finding is consistent with the systematic review by Maier et al., which identified difficulty initiating sleep as one of the most consistent risk factors for depression in older adults [7]. Similarly, Kerkez et al. reported that improvement in sleep quality was associated with a significant reduction in depressive symptoms among older adults [24]. Sleep disturbances have been associated with depression in previous studies, potentially through mechanisms involving neuroendocrine and inflammatory dysregulation; however, the cross-sectional design of the present study does not allow the direction of this association to be established.

Social isolation was also independently associated with depression in this study. Participants experiencing social isolation had approximately twice the odds of depression compared with socially connected individuals. Similar findings have been reported by Santini et al., who demonstrated that social disconnectedness was associated with increased symptoms of depression and anxiety among older adults [5]. Lin et al. also highlighted the importance of social relationships for psychological well-being among older adults [23]. Likewise, Banerjee et al. reported that infrequent social interactions and poor social engagement were significantly associated with depressive symptoms [25]. Social relationships provide emotional support, companionship, and a sense of belonging; therefore, reduced social participation may be associated with greater feelings of loneliness and helplessness, which may in turn be associated with a greater burden of depressive symptoms among older adults.

Widowhood was also independently associated with depression. Older adults who had lost their spouse were approximately twice as likely to experience depression compared to married participants. Similar associations between bereavement and depression have been documented by Brinda et al., Barik et al., Yaka et al., and Sengupta et al. [6,26-28]. The loss of a spouse may be associated with emotional distress, loneliness, reduced social support, financial insecurity, and difficulties in adapting to daily life, which may be related to depressive symptoms in later life.

Chronic morbidity was also independently associated with depression. This finding is supported by studies conducted by Sharma et al., Chauhan et al., Shaw et al., Mathew et al., and Bincy et al. [20,21,29-32], all of whom identified chronic illnesses as important factors associated with depression among older adults. Chronic diseases such as diabetes, hypertension, osteoarthritis, and cardiovascular disorders are often associated with persistent pain, functional limitations, frequent healthcare visits, and dependency, factors that may be associated with a greater burden of depressive symptoms. For anxiety, sleep difficulty again showed the strongest association. Participants with sleep problems had more than four times higher odds of anxiety. This finding is consistent with reports from Xie et al. and Raeisvandi et al., who observed significant associations between sleep disturbances and anxiety symptoms among older adults [13,19]. Sleep deprivation may be associated with increased autonomic arousal and emotional instability, which may partly explain its association with anxiety symptoms.

Social isolation was also independently associated with anxiety. Similar observations have been reported by Santini et al., who demonstrated longitudinal associations between social disconnectedness and anxiety symptoms [4,5]. Reduced social interaction may deprive older adults of emotional reassurance and practical support, thereby increasing feelings of insecurity and worry. Chronic morbidity was also significantly associated with anxiety. Comparable findings have been reported by Xie et al. and Raeisvandi et al. [13,19]. Chronic illnesses may be associated with greater uncertainty regarding future health, disability, and treatment outcomes, which may contribute to anxiety symptoms; however, the direction of this association cannot be established from the present cross-sectional data. Furthermore, the need for long-term medication use and healthcare expenditures may be associated with greater psychological distress.

The association observed between socioeconomic status and anxiety is also supported by previous studies. Xie et al., Brinda et al., Sengupta et al., Amu et al., and Rajkumar et al. reported that financial insecurity and poor economic conditions significantly increase vulnerability to anxiety and other psychological disorders among older adults [6,13,28,33-35]. However, the direction of association observed in this study differed from several previous studies and may reflect contextual socioeconomic differences, residual confounding, or reporting variations within the study population. One possible explanation is that stronger family and social support, including support associated with joint-family living, may buffer social isolation and psychological distress among some older adults from lower socioeconomic households. Conversely, economic hardship may be associated with reduced access to healthcare, lower social participation, and greater dependence on family members, which may adversely affect mental well-being.

The stress model revealed that social isolation showed the strongest independent association with stress. Socially isolated older adults had more than five-fold higher odds of experiencing stress compared with socially connected participants. Similar findings have been reported by Santini et al., Banerjee et al., Hosseini et al., and Liu et al., all of whom emphasized the protective role of social support and social participation in maintaining mental health among older adults [5,25,36,37]. Physical inactivity was also independently associated with stress in this study. This finding agrees with observations by Liu et al., Grover et al., de Oliveira et al., Lee et al., and Ku et al., who reported that physically active older adults experienced lower levels of depression, anxiety, and stress [37-41]. Physical activity has been associated with better cardiovascular fitness, sleep quality, self-esteem, and social engagement, as well as lower levels of inflammatory processes associated with psychological distress. Sleep difficulty and chronic morbidity remained significantly associated with stress after adjustment for other covariates. Similar associations have been reported by Behera et al., Raeisvandi et al., Sharma et al., Chauhan et al., and Hosseini et al. [8,19,21,29,36]. The cumulative burden of chronic illness and impaired sleep may be associated with lower resilience and coping ability and with greater levels of stress among older adults.

Interestingly, although female sex, older age, widowhood, and illiteracy demonstrated significant associations in bivariate analyses, these variables lost statistical significance after multivariable adjustment. Similar findings have been reported by Maier et al., Lin X et al., and Beniusiene et al., suggesting that the effects of these demographic factors may be mediated through more proximal determinants such as chronic disease burden, social isolation, sleep quality, and physical activity [7,23,33]. This finding highlights the potential importance of addressing modifiable factors associated with psychological distress rather than focusing solely on demographic characteristics. These findings are also consistent with previous evidence demonstrating that psychological well-being in later life is influenced by multiple social and health-related factors [23]. This study has important public health implications. The identified predictors - sleep difficulty, social isolation, chronic morbidity, widowhood, and physical inactivity - are potentially modifiable and can be identified during routine primary healthcare encounters. Community-based screening programs, older adult support groups, social engagement initiatives, physical activity promotion, and integrated management of chronic diseases may be useful components of strategies to address psychological distress among older adults. The findings support recommendations made by Jalali et al., Brinda et al., Zenebe et al., Behera et al., and Raeisvandi et al., who emphasized the need for early identification and targeted intervention strategies for geriatric mental health [3,6,8,14,19].

Limitations

This study has certain limitations that should be considered while interpreting the findings. First, as the study was conducted in rural areas of North India, the results may not be generalizable to urban populations or other regions with different sociocultural settings. Second, because information on depression, anxiety, stress, and related factors was collected through self-reported responses using the DASS-21 questionnaire, some participants may have underreported their symptoms due to stigma, poor awareness, or reluctance to discuss mental health issues. In addition, although the DASS-21 is a widely used and validated screening instrument, it is not a diagnostic tool, and its performance in older adults may differ from that in younger populations. Although the Hindi version was independently back-translated and pilot-tested among 50 older adults, formal psychometric validation and internal consistency assessment of the study-specific translation were not performed. Therefore, the possibility of some measurement error related to the translated instrument cannot be completely excluded. Third, although a multistage sampling design was used, a design-effect adjustment was not incorporated into the original sample-size calculation, and clustering was not explicitly accounted for in the statistical analysis using complex-survey or cluster-adjusted methods. Therefore, the potential impact of clustering on the precision of the estimates cannot be excluded. Fourth, the replacement of locked, unoccupied, or households without an eligible participant with an adjacent household, rather than revisiting the originally selected household, may have introduced selection bias by preferentially including more readily accessible participants and potentially underrepresenting socially isolated or homebound older adults. Fifth, sleep difficulty was assessed using a single self-reported yes/no item rather than a validated sleep assessment instrument. Although sleep duration was additionally recorded, the single-item measure may not comprehensively capture sleep quality, duration, severity, or specific sleep disorders and may have introduced measurement error or misclassification. Therefore, the observed associations between sleep difficulty and psychological symptoms should be interpreted with caution. Sixth, the cross-sectional design precludes establishing temporal directionality or causal relationships between the identified factors and psychological symptoms. Seventh, medication use was not independently assessed as a covariate in the multivariable models. Therefore, the possibility that unmeasured clinical factors or medication-related differences may have influenced some observed associations cannot be completely excluded.

## Conclusions

Depression, anxiety, and stress are significant mental health problems among older adults residing in rural North India. Sleep difficulty, chronic morbidity, social isolation, and physical inactivity were independently associated with psychological distress, while widowhood was independently associated with depression. These findings support the need for integrated geriatric mental health screening and community-based intervention strategies focused on potentially modifiable factors.
